# Association between complicated liver cirrhosis and the risk of hepatocellular carcinoma in Taiwan

**DOI:** 10.1371/journal.pone.0181858

**Published:** 2017-07-24

**Authors:** Tsung-Hsing Hung, Chih-Ming Liang, Chien-Ning Hsu, Wei-Chen Tai, Kai-Lung Tsai, Ming-Kun Ku, Jiunn-Wei Wang, Kuo-Lun Tseng, Lan-Ting Yuan, Seng-Howe Nguang, Shih-Cheng Yang, Cheng-Kun Wu, Pin-I Hsu, Deng-Chyang Wu, Seng-Kee Chuah

**Affiliations:** 1 Division of Gastroenterology, Department of Internal Medicine, Dalin Tzu Chi Hospital, Buddhist Tzu Chi Medical Foundation, Chia-Yi, Taiwan; 2 School of Medicine, Tzu Chi University, Hualien, Taiwan; 3 Division of Hepato-gastroenterology; Department of Internal Medicine, Kaohsiung Chang Gung Memorial Hospital, Kaohsiung, Taiwan; 4 Department of Pharmacy, Kaohsiung Chang Gung Memorial Hospital, Kaohsiung, Taiwan; 5 School of Pharmacy, Kaohsiung Medical University, Kaohsiung, Taiwan; 6 Chang Gung University, College of Medicine, Kaohsiung, Taiwan; 7 Division of Colon and Rectal Surgery, Department of Surgery, Kaohsiung Chang Gung Memorial Hospital, Kaohsiung, Taiwan; 8 Division of Gastroenterology; FooYin University Hospital, Pin-Tung, Taiwan; 9 Division of Gastroenterology, Department of Internal Medicine, Kaohsiung Municipal Ta-Tung Hospital, Kaohsiung Medical University Hospital and Center for Stem Cell Research, Kaohsiung Medical University, Kaohsiung, Taiwan; 10 Divisions of Gastroenterology, Yuan General Hospital, Kaohsiung, Taiwan; 11 Division of Gastroenterology; Pin-Tung Christian Hospital, Pin-Tung, Taiwan; 12 Division of Gastroenterology, Department of Internal Medicine, Kaohsiung Veterans General Hospital, National Yang-Ming University, Kaohsiung, Taiwan; Yonsei University College of Medicine, REPUBLIC OF KOREA

## Abstract

Hepatic encephalopathy, ascites, and variceal bleeding are the three major complications of cirrhosis. It is well known that cirrhosis is the most important risk factor of hepatocellular carcinoma (HCC). However, little is known about whether the severity of liver cirrhosis has an effect on the incidence of HCC. This population-based cohort study aimed to explore the association between complicated cirrhosis and HCC, and identify the risk factors of HCC in patients with complicated cirrhosis. Data of the years 1997–2011 were extracted from the National Health Insurance Research Database of Taiwan. A total of 2568 patients with complicated cirrhosis without HCC at baseline were enrolled. After propensity score matching, another 2568 patients with non-complicated cirrhosis were included. Hazards Cox regression analysis by using a competing risk regression model to control for possible confounding factors was utilized to estimate the association of the complications of liver cirrhosis with the risk of HCC. We observed by using competing risk analysis that the adjusted hazard ratio (HR) for developing HCC during the follow-up period after the initial hospitalization was higher among the patients with baseline complicated cirrhosis than in those with uncomplicated cirrhosis (HR, 1.23; 95% confidence interval, CI, 1.10–1.37, p<0.001). Additionally, older patients (HR, 1.01; 95% CI, 1.01–1.02, p<0.001), males (HR, 0.84; 95% CI, 0.74–0.96, p = 0.009), and patients with alcohol-related cirrhosis (HR, 1.93; 95% CI, 1.65–2.26, p<0.001) had a statistically significant difference in the incidence of HCC. In conclusion, complicated liver cirrhosis is associated with a higher risk of HCC in Taiwan compared with cirrhosis without complications.

## Introduction

Hepatocellular carcinoma (HCC) represents 70%-85% of primary liver malignancies [1,2]. With abdominal sonography, we can screen for HCC and perform surveillance to detect and treat tumors in the early stages. It is well known that most cases of HCC are associated with cirrhosis regardless of the etiology [3–8]. The 5-year cumulative risk of developing HCC for patients with cirrhosis ranges between 5% and 30% [3, 4]. The mortality of patients with cirrhosis with cirrhosis-related complications is high and many of them die before they develop HCC [9–11]. However, it has not been reported whether the complications of cirrhosis are associated with the development of HCC. It is interesting and noteworthy to better understand the association between the two by using competing risk analysis model appropriately. It is well known that male patients have a higher risk of HCC than female patients do, and the risk of cirrhosis is also higher in male patients [12–15]. Since cirrhosis is the most important risk factor of HCC, it will be interesting to know the difference in the incidence of HCC between the sexes in patients with cirrhosis, especially in those with complications.

This population-based cohort study aimed to explore the association between complicated cirrhosis and HCC, and identify the risk factors of HCC in patients with complicated cirrhosis.

## Materials and methods

### Compliance with ethical requirements and database

This protocol was approved by the institutional review board and the Ethics Committee of Chang Gung Memorial Hospital and Kaohsiung Medical University Hospital, Kaohsiung, Taiwan. The Ethics Committee waived the requirement for informed consents for this study, and the data were analyzed anonymously. The claims data for our study were collected from Taiwan’s National Health Insurance Research Database (NHIRD) for the years 1997–2011. A cohort dataset of one million randomly selected individuals was used, which was released by the National Health Research Institutes. The released dataset for this study did not include the private data of the patients or their health care providers. The details of the database have been described in previous studies [16–18]. Kaohsiung Medical Center is one of the sites of the Collaboration Center of Health Information Application, Ministry of Health and Welfare. The data analyst of our study was one of the staff members and she analyzed the health care data, which included the enrollment files and claims data.

### Study sample

**Fig 1:**
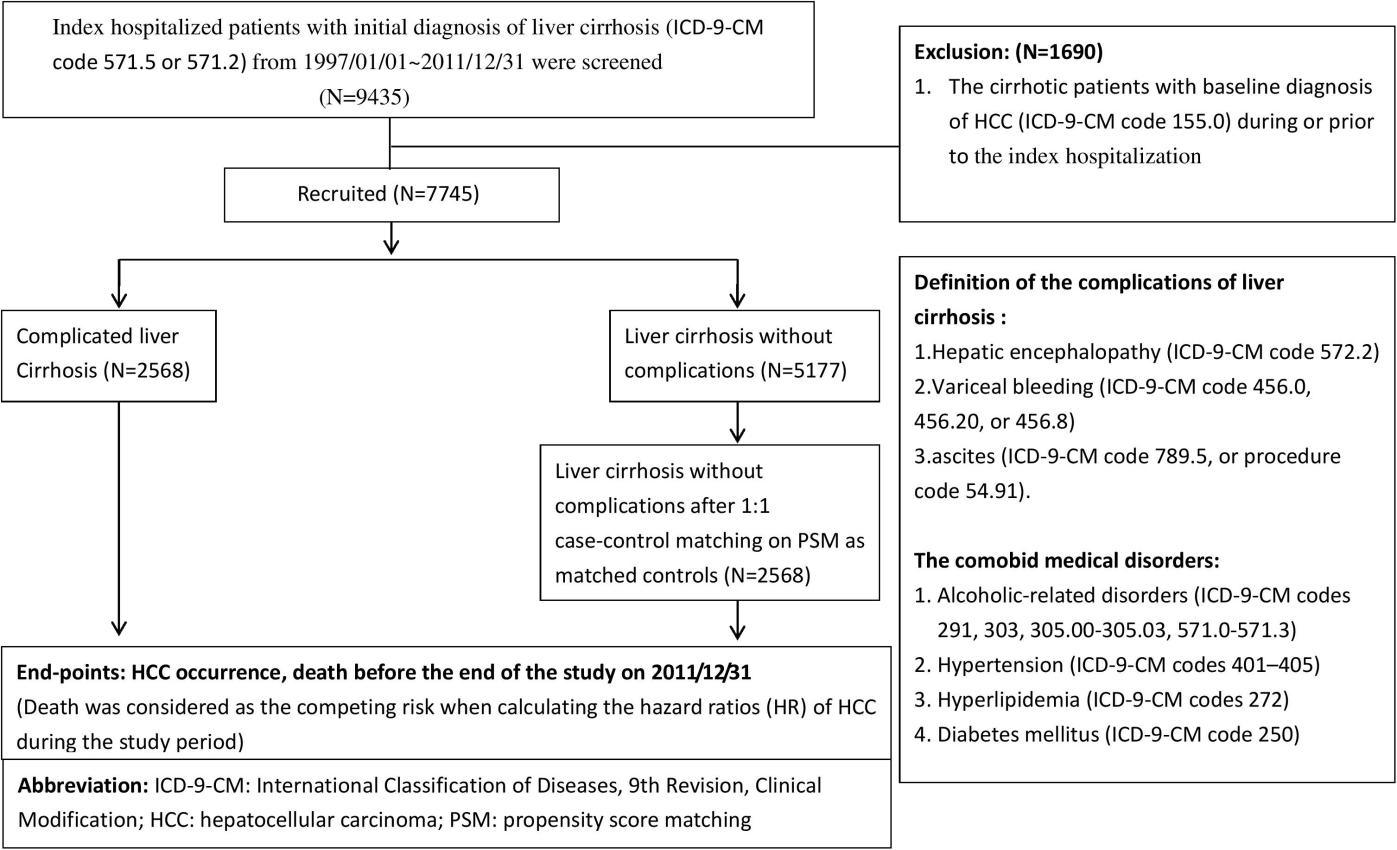
The schematic flowchart of study design.

A schematic flowchart of the study design is shown in Fig 1. Data were extracted from the NHIRD using the International Classification of Diseases, 9th Revision, Clinical Modification (ICD-9-CM) code 571.5, or code 571.2 to screen for index hospitalization of patients with an initial diagnosis of liver cirrhosis who were discharged between January 1, 1997 and December 31, 2011 (n = 9435). All these patients were followed up until December 31, 2011. In this population-based cohort study, a total of 1690 patients with cirrhosis with baseline diagnosis of HCC (ICD-9-CM code 155.0) during or prior to the index hospitalization were excluded.

Subsequently, a total of 7745 patients with cirrhosis were recruited. They were then divided into two groups: the complicated liver cirrhosis and uncomplicated liver cirrhosis groups (n = 2568 and n = 5177, respectively). Complicated liver cirrhosis was defined as cirrhosis with cirrhosis-related complications. The complications of liver cirrhosis in our study were hepatic encephalopathy (HE) (ICD-9-CM code 572.2), variceal bleeding (VB) (ICD-9-CM code 456.0, 456.20, or 456.8), and ascites (ICD-9-CM code 789.5, or procedure code 54.91).

We performed one-to-one case-control match using propensity score matching (PSM) to avoid the interference of confounding factors. The factors for PSM were age, sex, year of enrollment, alcohol-related, diabetes mellitus, hypertension, and hyperlipidemia. After PSM, 2568 patients with uncomplicated cirrhosis were enrolled in this study as matched controls. Death was considered the competing risk when calculating the hazard ratios (HR) of HCC during the study period. The comorbidities included alcohol-related disorders (ICD-9-CM codes 291, 303, 305.00–305.03, and 571.0–571.3), hypertension (ICD-9-CM codes 401–405), hyperlipidemia (ICD-9-CM code 272), and diabetes mellitus (ICD-9-CM code 250).

### Statistical analyses

The baseline characteristics were compared using Student t test and chi-square analyses for continuous and nominal variables, respectively. Hazards Cox regression by using competing risk regression model was used to control possible confounding factors. The values of P < 0.05 were considered statistically significant. The analyses were performed using the statistical software package SAS version 9.3 (SAS Institute Inc., Cary, NC, 2013)

## Results

Table 1 shows the distribution of the demographic characteristics and comorbidities at baseline for the two groups of patients with cirrhosis with and without cirrhosis-related complications. Of the 2568 patients with complicated liver cirrhosis, the mean age was 57.1 ± 15.4 years and 1825 (71.0%) were males. Of the2568 patients with uncomplicated liver cirrhosis, the mean age was 57.6 ± 15.6 years and 1784 (69.5%) were males. Seven-hundred and sixty six patients (28.9%) in thecomplicated liver cirrhosis group and 743 (29.9%) in the uncomplicated group had alcohol-related cirrhosis. There were no significant differences between the two groups in the incidence of other comorbidities such as diabetes mellitus (27.1% vs. 25.4%, p = 0.173), hyperlipidemia (8.0% vs. 8.2%, p = 0.838), and hypertension (23.5% vs. 22.9%, p = 0.60). Table 2 demonstrates the outcomes in male and female patients. Among the 5136 patients with cirrhosis, 1269 patients developed HCC during the follow up period with higher proportion in men (n = 863, 68.0%). At the same time, 1810 patients died during the follow up period and the mortality was higher in men (n = 1243, 68.7%). The cumulative incidences of HCC in the complicated and non-complicated cirrhosis groups were 35.6% and 29.1% in ten years, respectively (P = 0.002). **I**n the complicated cirrhosis group, 40.3% (1036/2568) patients died before developing HCC, while 30.1% (774/2568) died before developing HCC in the non-complicated cirrhosis group. The percentages of patients who developed HCC before death in the complicated and non-complicated cirrhosis groups were 26.4% (678/2568) and 23.0% (591/2568), respectively.

**Table 1:** Demographic characteristics of complicated and uncomplicated cirrhotic patients.

	Complicated cirrhotic patients (n = 2568)	Uncomplicated cirrhotic patients (n = 2568)	*P* value
Male, n (%)	1825 (71.1)	1825 (71.1)	0.211
Age, years	57.1± 15.4	54.1± 15.6	0.206
**Comorbid conditions**			
Acute myocardial infarction, n (%)	24(0.93)	33(1.29)	0.231
Congestive heart failure, n (%)	208(8.10)	199(7.75)	0.642
Peripheral vascular disease	34(1.32)	48(1.87)	0.119
Connective tissue disorder	33(1.29)	30(1.17)	0.704
HIV	3(0.12)	6(0.23)	0.317
Alcohol-related, n (%)	766 (29.8)	743 (28.9)	0.481
DM, n (%)	696 (27.1)	653 (25.4)	0.173
Hyperlipidemia, n (%)	206 (8.0)	210 (8.2)	0.838
Hypertension, n (%)	604 (23.5)	588 (22.9)	0.597
**Year of enrollment**			0.704
1997 1998 1999 2000 2001 2002 2003 2004 2005 2006 2007 2008 2009 2010 2011	69(2.69%) 67(2.61%) 67(2.61%) 62(2.41%) 97(3.78%) 98(3.82%) 154(6.00%) 178(6.93%) 271(10.55%) 245(9.54%) 246(9.58%) 258(10.05%) 239(9.31%) 290(11.29%) 227 (8.84%)	65(2.53%) 69(2.69%) 64(2.49%) 76(2.96%) 101(3.93%) 109(4.24%) 156 (6.07%) 167(6.50%) 256(9.97%) 230(8.96%) 229(8.92%) 243(9.46%) 257(10.01%) 270(10.51%) 276(10.75%)	

**Abbreviations:** DM, diabetes mellitus: HIV: human immunodeficiency virus

**Table 2:** Outcomes of cirrhotic patients with difference sex.

Characteristics	Complicated cirrhotic patients (n = 2568)				Uncomplicated cirrhotic patients (n = 2568)				
	male (n = 1825)	%	female (n = 743)	%	male (n = 1810)		%	female (n = 759)	%
**Endpoint**									
HCC*	470	25.8	208	28.0	393		22.0	198	25.3
Death**	719	39.4	317	42.7	524		29.4	250	31.9

**Abbreviations:** HCC: hepatocellular carcinoma

* Among the 5136 cirrhotic patients, 1269 patients encountered the occurrence of the HCC during the follow up period with male predominance (n = 863, 68.0%).

**At the same time, 1810 patients died during the follow up period and were also male predominance (n = 1243, 68.7%).

After adjusting for gender, age, and medical comorbidities, patients with complicated cirrhosis had a higher risk of HCC than those with uncomplicated cirrhosis (HR, 1.23; 95% confidence interval, CI, 1.11–1.37, p<0.001). Additionally, older patients (HR, 1.01; 95% CI, 1.01–1.02, p<0.001), males (HR, 0.84; 95% CI, 0.74–0.96, p = 0.009), and patients with alcohol-related cirrhosis (HR, 1.93; 95% CI, 1.65–2.26, p<0.001) had a statistical significant difference in the incidence of HCC (Table 3)

**Table 3:** Adjusted hazard ratios for the occurrence of hepatocellular carcinoma (HCC) during the follow-up period following first hospitalization by using competing risk analysis.

Variable	Hazard ratio	95% confidence interval	*P* value
Age	1.01	1.01–1.02	<0.001
Male	0.84	0.74–0.96	0.001
Alcohol-related	1.93	1.65–2.26	<0.001
Diabetes mellitus	0.90	0.98–1.01	0.403
Hypertension	1.16	1.00–1.35	0.046
Hyperlipidemia	1.26	0.99–1.59	0.061
With complications*	1.23	1.10–1.37	<0.001
Year of enrollment	0.99	0.98–1.01	0.403

**Abbreviations:** HCC, hepatocellular carcinoma

* hepatic encephalopathy, esophageal variceal bleeding, or hepatic encephalopathy

**Fig 2:**
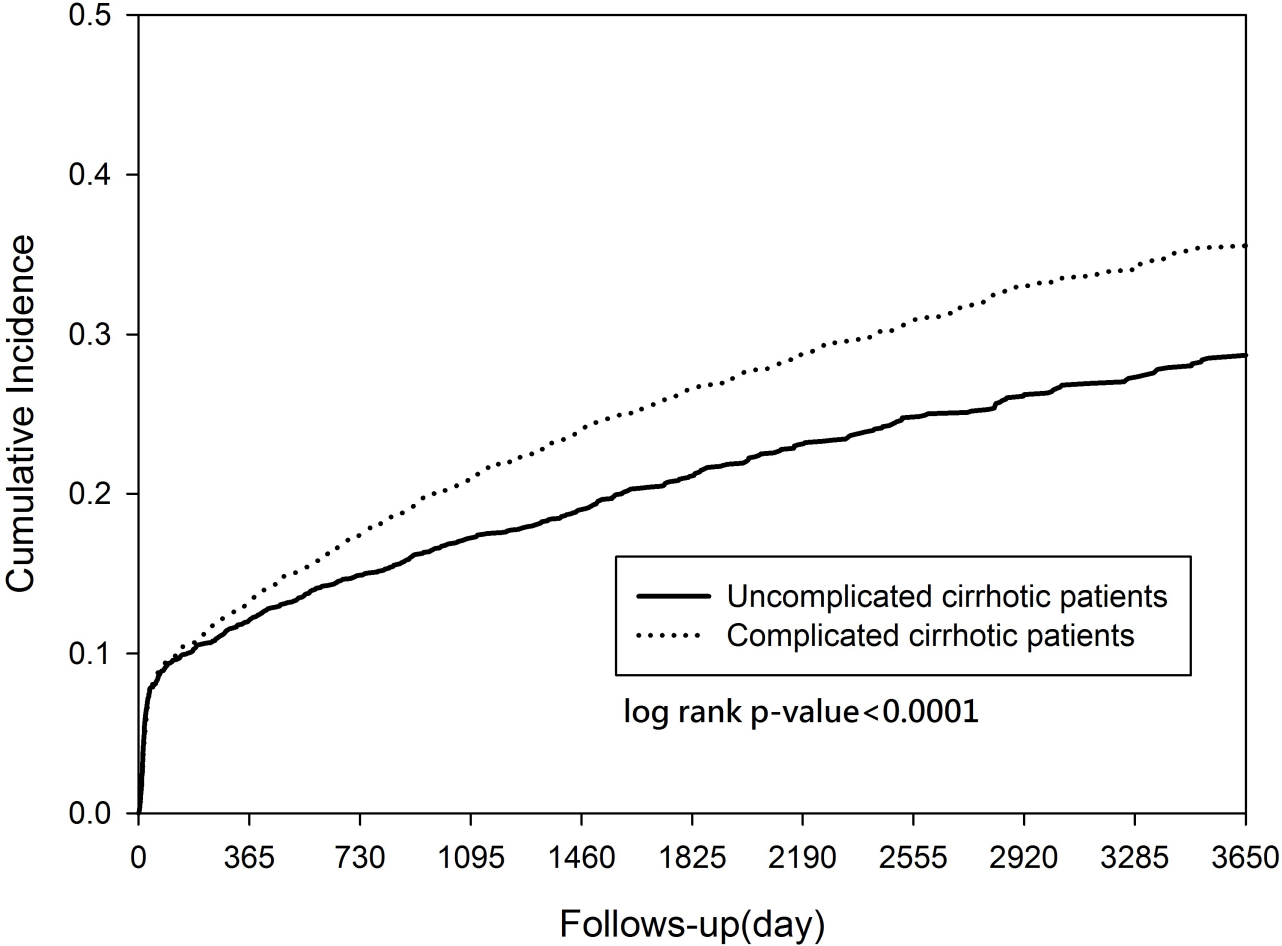
Cumulative incidence curves for the hepatocellular carcinoma (HCC) in complicated and uncomplicated cirrhotic patients.

The cumulative incidences of HCC are presented in Fig 2. The cumulative incidence curves for HCC showed that the incidence was much higher in patients with complicated cirrhosis than in those with uncomplicated cirrhosis (p<0.001).

## Discussion

Although it is well known that cirrhosis is the most important risk factor of HCC [3–8], little is known about whether the severity of cirrhosis can affect the development of HCC in patients with cirrhosis. Certain studies have established the correlation between the degree of liver fibrosis and HCC [19, 20]. Cirrhotic patients with complications are in decompensated states. In this study, we evaluated the risk of developing HCC in different severities of liver cirrhosis. Liver cirrhosis with complications is reflective of the severity of liver disease, and is correlated with higher incidence of HCC.

Previous studies have showed that the annual incidence of HCC in patients with cirrhosis was about 3–5 percent per year, which was the same as that in the current study [21–23]. However, the mortality in patients with cirrhosis is high, especially in those with cirrhosis-related complications. Therefore, it may not be easy to estimate the actual risk of developing HCC in patients with cirrhosis because many of them die before they develop HCC. In clinical practice, physicians usually pay more attention to the cirrhosis-related complications in these patients due to the high mortality associated with them. Rationally, the presence of cirrhosis-related complications represents a more advance stage of the disease. Nevertheless, there are only a few studies on the association between cirrhosis-related complications and the risk of HCC. Furthermore, there may be many other confounding factors during the process of data analysis. In the current study, we performed PSM using one-to-one case-control matching to minimize bias. Finally, we included 2568 patients with uncomplicated cirrhosis as matched controls. Since death was considered as the competing risk when calculating the hazards ratio of HCC, a competing risk analysis model was used to assess the actual risk of HCC in these patients.

Among a total of 1269 cirrhosis patients with HCC, 863(68.0%) were male, which is in accordance with the current literature. Previous reports suggest a male/female ratio in patients with HCC to be between 2:1 and 4:1 in almost all populations [14]. In this study, about 70% of patients with cirrhosis were also males. This can explain the male predominance in HCC. On the other hand, interestingly, the current study observed that the risk of HCC in female patients with cirrhosis is not lower than that in male patients with cirrhosis, after adjusting for other factors as shown in Table 3 (HR: 0.85; 95% CI 0.75–0.97). This may suggest that the male predominance in HCC may just be because of the higher incidence of liver cirrhosis in male patients. In Taiwan, the vaccination program against hepatitis B virus (HBV) infection was initiated in 1984, which has reduced the incidence of HCC in children and adolescents successfully [24]. Huang et al. later reported that the hazards ratio of developing HCC decreased with age in HBsAg-seropositive men but increased with age in anti-HCV–seropositive women [25]. Further studies are needed to explain these observations. These findings remind physicians to closely monitor the patients with cirrhosis for HCC, regardless of the gender.

The adjusted hazard ratios for HCC during the follow-up period following the first hospitalization by using competing risk analysis showed that alcohol consumption is an important risk factor among these patients (HR, 1.93; 95% CI, 1.65–2.26, p<0.001). This finding is in accordance with reports in the literature that alcohol consumption is an important risk factor for HCC, especially in patients with liver cirrhosis [26]. It is well established that the amount of alcohol consumption is proportionally correlated to the degree of liver injury and, hence, of developing cirrhosis and HCC [27]. Turati and colleagues reported that consumption of three or more drinks per day resulted in 16% higher risk of liver cancer and consumption of six or more drinks per day resulted in 22% higher risk [28].

This study has some limitations. First, we were unable to assess the severity of liver cirrhosis as measured by validated scoring systems such as MELD score or Child-Pugh score because we could not identify laboratory information from the dataset. The drugs used in these patients could not be identified, either. This is the major limitation of our study. Second, we could not distinguish between the etiologies of non-alcoholic liver cirrhosis in this study and some personal data such as body weight were not identifiable. However, the strength of our study is that we have demonstrated that patients with complicated cirrhosis had a higher risk of HCC, implying that the more advanced stages of liver disease carry higher risk of HCC. Further large-scale clinical studies are required to clarify the actual association of the severity of liver cirrhosis and HCC. Third, the surveillance procedures of HCC could not be identified in this study and the diagnosis of HCC is based on ICD-9 coding. Fourth, to increase the accuracy of diagnosis, we screened for hospitalized patients with an initial diagnosis of liver cirrhosis. However, the patients who are relatively “healthy” do not need admission. Indeed, the severity of cirrhosis in the patients in this study was greater than that in outpatients, and this may have led to selection bias. Otherwise, HCC was one of the reasons for admission and many patients with cirrhosis were admitted for the treatment of HCC. This could explain why there was such a high initial rate of HCC in this study.

In conclusion, complicated liver cirrhosis is associated with a higher risk of HCC in Taiwan compared to those without complications.

## Supporting information

S1 Dataset This file provides the data of the manuscript. (XLS)
